# Regional Vulnerability Indices in Youth With Persistent and Distressing Psychoticlike Experiences

**DOI:** 10.1001/jamanetworkopen.2023.43081

**Published:** 2023-11-13

**Authors:** Nicole R. Karcher, Hailey Modi, Peter Kochunov, Si Gao, Deanna M. Barch

**Affiliations:** 1 Washington University in St Louis School of Medicine, St Louis, Missouri; 2 Maryland Psychiatric Research Center, University of Maryland School of Medicine, Baltimore, Maryland; 3 Department of Psychiatry, University of Texas Health Science Center, Houston; 4 Student, Washington University in St Louis, St Louis, Missouri

## Abstract

**Importance:**

Distressing and persistent psychoticlike experiences (PLEs) in youth are associated with greater odds of developing psychiatric conditions in adulthood. Despite this risk, it is unclear whether early PLEs show similar brain patterns compared with adults with psychiatric and neurologic conditions.

**Objective:**

To examine the degree to which persistent and distressing PLEs exhibit neural metrics that show similarity to adults with chronic psychiatric and neurologic conditions.

**Design, Setting, and Participants:**

This cohort study used Adolescent Brain Cognitive Development (ABCD) Study examining the persistence and distress associated with PLEs across the first 3 waves of data with baseline structural magnetic resonance imaging data. Analyzed data were collected between September 1, 2016, and September 27, 2021. Children were recruited from 21 research sites across the US.

**Exposures:**

Psychoticlike experiences were assessed using the Prodromal Questionnaire–Brief Child Version, and participants were categorized into groups based on the persistence and distress associated with PLEs.

**Main Outcomes and Measures:**

Cortical and subcortical regional vulnerability indices (RVIs) were created by quantifying the similarity of participants’ baseline neuroimaging measures to the expected patterns found in adult neuropsychiatric samples. The PLE groups were compared on the following RVI cortical and subcortical metrics: schizophrenia spectrum disorders, bipolar disorder, major depressive disorder, Parkinson disease, Alzheimer disease, and metabolic diseases.

**Results:**

Analyses examined PLE groups created from 8242 children in the ABCD sample (52.5% male; mean [SD] age, 9.93 [0.63] years; and 56.3% White), including persistent distressing PLEs (n = 329), transient distressing PLEs (n = 396), persistent nondistressing PLEs (n = 234), transient nondistressing PLEs (n = 390), and low distressing PLEs (n = 6893) groups. Participants with persistent or transient distressing PLEs broadly showed increased subcortical RVI scores across most RVI metrics, with persistent distressing PLEs additionally showing increased scores for cortical RVI metrics. The greatest effect sizes were found for persistent distressing PLEs with cortical RVI-schizophrenia spectrum disorders (β estimate, 1.055; 95% CI, 0.326-1.786) and RVI-Alzheimer disease (β estimate, 2.473; 95% CI, 0.930-4.018).

**Conclusions and Relevance:**

In this cohort study of ABCD participants, the findings suggest that especially the persistent distressing PLEs in children were associated with neural metrics resembling those observed in adults with severe psychiatric and neurologic conditions. These findings support the potential use of brain-based risk scores for early identification and precision medicine approaches in the assessment of PLEs.

## Key Points

**Question:**

Do youth with early psychoticlike experiences (PLEs) show structural brain patterns similar to adults with chronic mental and physical illness?

**Findings:**

In this cohort study using Adolescent Brain Cognitive Development Study data, the persistence and distress associated with PLEs were examined in 8242 youth aged 9 to 13 years, finding evidence that it was especially the neural metrics for youth experiencing persistent distressing PLEs that showed greater similarity to neural metrics for adults with chronic mental and physical health conditions. The largest effect sizes were found for persistent distressing PLEs with brain-based risk metrics for schizophrenia spectrum disorders and Alzheimer disease.

**Meaning:**

The similarity-based index differentiated distressing PLE groups from low PLEs in otherwise healthy children, indicating risk scores that are based on findings of large neuropsychiatric studies, which may provide an avenue for precision medicine efforts.

## Teaser

This cohort study examines the use of cortical and subcortical regional vulnerability indices to estimate the degree to which persistent and distressing psychoticlike experiences in children exhibit neural metrics that show similarity to adults with chronic psychiatric and neurologic conditions.

## Introduction

Psychoticlike experiences (PLEs) are unusual thought content and perceptual aberrations that fit within the extended psychosis phenotype.^1,2^ Psychoticlike experiences are associated with a higher risk for developing a schizophrenia spectrum disorder (SSD) (odds ratio [OR], 16.4)^3^ and higher odds of other mental illnesses, including bipolar disorder (BD) (OR, 2.0) and major depressive disorders (MDDs) (OR, 1.3).^4^ Distressing and persistent PLEs are associated with impairments in functioning compared with nondistressing or transient PLEs.^2^ Psychoticlike experiences are also linked to chronic body illnesses, including diabetes, hypertension, and metabolic dysregulation,^5^ and higher lifetime rates of Alzheimer disease (AD) and Parkinson disease (PD).^6^ The substantial increase of mental health risk, including higher rates of psychosis, calls for early identification of relevant PLEs in middle childhood and early adolescence before the onset of the psychotic symptoms.

We are part of a group developing biomarkers for the assessment of early disease risk by quantifying the similarity of an individual’s brain to the pattern of effect sizes reported by large and inclusive meta-analytic studies. This group developed the regional vulnerability index (RVI)^7^ that represents the degree of deviation from healthy brains in the direction of disease, as defined by the largest studies in the corresponding illnesses performed by the Enhancing Neuro Imaging Genetics Meta Analyses (ENIGMA) consortium.^8^ Previous work using the RVI has observed that the participants in the Adolescent Brain Cognitive Development (ABCD) Study with a family history of schizophrenia and more stressful life experiences exhibited structural brain patterns that showed greater similarity to adults with SSDs (RVI-SSDs).^9^ The RVI approaches are important for child and adolescent populations that do not anticipate large effect-size neural deficits, whereby RVI can quantify deviations in the direction of disease. Herein, we examined whether youth with distressing and/or persistent PLEs have significant increases in RVI-SSDs. We further studied the specificity of these patterns by calculating the RVI for other clinical states, including BD, MDD, PD, AD, and metabolic diseases (METs). It is expected that, although brains with persistent and/or distressing PLEs would show some similarity to the brains from these other clinical states, the greatest similarity would be found for mental health conditions, including SSDs, BD, and MDD. Parkinson disease, AD, and MET were included to examine specificity, as it was expected that these conditions would have smaller effect sizes given the generally later onset (PD, AD) and reduced phenotypic proximity (MET) of these conditions.

## Methods

### Participants

The ABCD Study is a large-scale study tracking children aged 9 to 10 years recruited from 21 research sites across the US, with the data used in the analyses collected between September 1, 2016, and September 27, 2021.^10^ In this cohort study, we included 3 waves of data: baseline (n = 11 878), 1-year follow-up (n = 11 235), and 2-year follow-up (n = 10 414) for the creation of PLE groups. Regional vulnerability index scores were created using baseline structural magnetic resonance imaging (MRI) data. The eMethods in Supplement 1 provides studywide exclusion criteria. All procedures were approved by a central institutional review board at the University of California, San Diego. All parents and children provided written informed consent (parents) and assent (children). Participants received financial compensation. This report followed the Strengthening the Reporting of Observational Studies in Epidemiology (STROBE) reporting guideline.

### Measures

To measure PLEs, participants completed the previously validated Prodromal Questionnaire**–**Brief Child Version (PQ-BC).^1^ After participants answered each item for the PQ-BC (yes/no), they were asked whether the experience bothered them (yes/no). If participants indicated that an item bothered them, they were asked to rate their distress on a scale from 1 to 5. Consistent with previous research,^1^ PQ-BC distress scores were calculated as the total number of 21 questions indicating distress weighted by distress rating. The PQ-BC nondistress scores were calculated as the total number of 21 questions not indicating distress.

Given that persistence cannot be measured dimensionally in this sample, 5 groups were created to examine persistence and distress (eTable 1 in Supplement 1), as has been done in previous research.^2^ Groups were characterized by whether they met the high symptom threshold (≥1.96 SDs above the mean) or the low symptom threshold (≤0.50 SDs above the mean) on PQ-BC scores for each of the 3 assessment waves. The 5 groups were defined as follows: (1) persistent distressing PLEs (n = 329) met the high symptom threshold for PQ-BC distress scores for 2 or more assessment waves, (2) transient distressing PLEs (n = 396) met the high symptom threshold for PQ-BC distress scores for 1 assessment wave and the low symptom threshold for the other 2 waves, (3) persistent nondistressing PLEs (n = 234) met the high symptom threshold for PQ-BC nondistress scores for 2 or more assessment waves, (4) transient nondistressing PLEs (n = 390) met the high symptom threshold for PQ-BC nondistress scores for 1 assessment wave and the low symptom threshold for the other 2 waves, and (5) low distressing PLEs (n = 6893; ie, the healthy control reference group) met the low symptom thresholds for all waves.

### Structural MRI

Structural MRI measures included all baseline metrics of cortical thickness and subcortical volume. Structural neuroimaging processing was completed using FreeSurfer, version 5.3.0 (Martinos Center for Biomedical Imaging) through standardized processing pipelines.^11^ Analyses included participants with thickness and volume data that passed ABCD Study quality control recommendations. All 34 cortical thickness structures from the Desikan atlas and all 9 subcortical volume structures from the Desikan atlas were included in the creation of RVI metrics. Data were initially separated by hemisphere and were averaged to obtain a bilateral value.

### Regional Vulnerability Index

We calculated cortical and subcortical RVIs using the RVIpkg package in R.^7^ Following previous research,^12^ the RVI was a measure quantifying the similarity between each participant’s normalized neuroimaging measures and the expected pattern of differences from a community control group for a given illness metric (eg, SSDs) derived from meta-analyses. Following previous work,^9^ to create these scores for all observations for each neuroimaging feature, covariates (age, sex at birth, race and ethnicity, and overall intracranial volume) were regressed out using linear regression. All covariates were chosen due to associations between these characteristics and the mental and physical health conditions for which the RVI metrics were created. Residuals were extracted from this model, inverse-normal transformed, and *z* transformed. We then computed the dot product between a vector of the normalized residuals of all participants in the current sample (ie, a *z* score using the mean [SD] from the control group of the current sample) for each neural metric and a vector of effect sizes across neural metrics reported by ENIGMA for a particular disease class (eg, SSDs, BD), with separate estimates for cortical and subcortical regions (eTable 2 in Supplement 1 provides more information about characteristics of the studies used to calculate RVI scores).

### Statistical Analysis

We used linear mixed-effects models implemented in the lme4 package in R.^13^ All analyses modeled family unit and research site as random intercepts. Linear mixed-effects models analyzed each RVI score individually (ie, separate models for SSDs, BD, MDD, PD, AD, and MET) as the outcome, with PLE group status as the fixed effect (as mentioned above, age, sex at birth, race and ethnicity, and overall intracranial volume were regressed out in the creation of RVI scores). Models were false discovery rate (FDR) corrected^14^ for multiple comparisons across the 24 cortical RVI models and across all 24 subcortical RVI models. Models were considered significant if the FDR-corrected value was *P* < .05. Significance testing was 2-tailed and unpaired.

## Results

Analyses examined PLE groups created from 8242 children in the ABCD Study sample (52.5% male; 47.5% female; mean [SD] age, 9.93 [0.63] years; and 56.3% White). Table 1 reports all demographic characteristics for each of the PLE groups.

**Table 1.** Group Characteristics

Characteristic	Participants by PLE group, No. (%)				
	Persistent distressing (n = 329)	Transient distressing (n = 396)	Persistent nondistressing (n = 234)	Transient nondistressing (n = 390)	Low (n = 6893)
Sex					
Female	160 (48.6)	220 (55.6)	81 (34.6)	172 (44.1)	3279 (47.6)
Male	169 (51.4)	176 (44.4)	153 (65.4)	218 (55.9)	3614 (52.4)
Race and ethnicity^a^					
Asian	1 (0.3)	106 (26.8)	5 (2.1)	5 (1.3)	170 (2.5)
Black	83 (25.2)	99 (25.0)	75 (32.1)	100 (25.6)	652 (9.5)
Hispanic	91 (27.7)	50 (12.6)	48 (20.5)	71 (18.2)	1246 (18.1)
White	120 (36.5)	137 (34.6)	81 (34.6)	161 (41.3)	4144 (60.1)
Multiracial/multiethnic	34 (10.3)	4 (1.0)	25 (10.7)	53 (13.6)	681 (9.9)
Age, mean (SD), y	9.81 (0.62)	9.84 (0.62)	9.90 (0.61)	9.86 (0.61)	9.88 (0.62)
Distressing PLEs, mean (SD)					
Baseline	32.85 (19.44)	19.43 (19.05)	16.98 (12.12)	9.51 (9.49)	12.78 (53.79)
1-y Follow-up	48.82 (119.49)	54.52 (206.80)	27.56 (111.36)	60.82 (224.31)	176.95 (372.98)
2-y Follow-up	87.38 (238.86)	56.47 (206.45)	40.56 (168.90)	166.63 (365.91)	389.43 (482.97)
Nondistressing PLEs, mean (SD)					
Baseline	3.42 (2.78)	2.14 (2.31)	5.91 (3.02)	2.96 (3.16)	4.53 (53.15)
1-y Follow-up	18.80 (121.99)	46.55 (208.11)	18.68 (111.99)	55.99 (225.27)	171.57 (375.32)
2-y Follow-up	67.25 (243.69)	46.97 (208.02)	34.13 (169.82)	163.29 (367.30)	386.01 (485.65)

Abbreviation: PLEs, psychoticlike experiences.

^a^ Race and ethnicity is caregiver reported and categorized as in the National Institute on Drug Abuse. The multiracial/multiethnic category is 1 of the 5 categories created by the Adolescent Brain Cognitive Development (ABCD) Study and comprises everyone who did not fit into Asian, Black, Hispanic, or White groups (eg, generally multiracial/multiethnic but also including Native American/Alaska Native, Native Hawaiian or Other Pacific Islander, or individuals selecting other).

In comparison with the low PLE group, the persistent distressing PLE group showed higher cortical RVI-SSD (Figure; Table 2) (β estimate, 1.055; 95% CI, 0.326-1.786; *t* = 2.833; FDR *P* = .04; for subcortical SSD-RVI: β estimate, 0.116; 95% CI, 0.010-0.221; *t* = 2.152; FDR *P* = .06). Participants with persistent distressing PLEs also showed an increase in RVI for BD, MDD, PD, AD, and MET (all cortical and subcortical: *t* ≥ 2.456; FDR *P* < .05; for cortical MDD-RVI: β estimate, 0.115; 95% CI, 0.013-0.218; *t* = 2.215; FDR *P* = .11) (Figure; Table 2). Of these findings, the greatest effect sizes were found for persistent distressing PLEs with cortical RVI for SSD (β estimate, 1.055; 95% CI, 0.326-1.786) and AD (β estimate, 2.473; 95% CI, 0.930-4.018).

**Figure.**
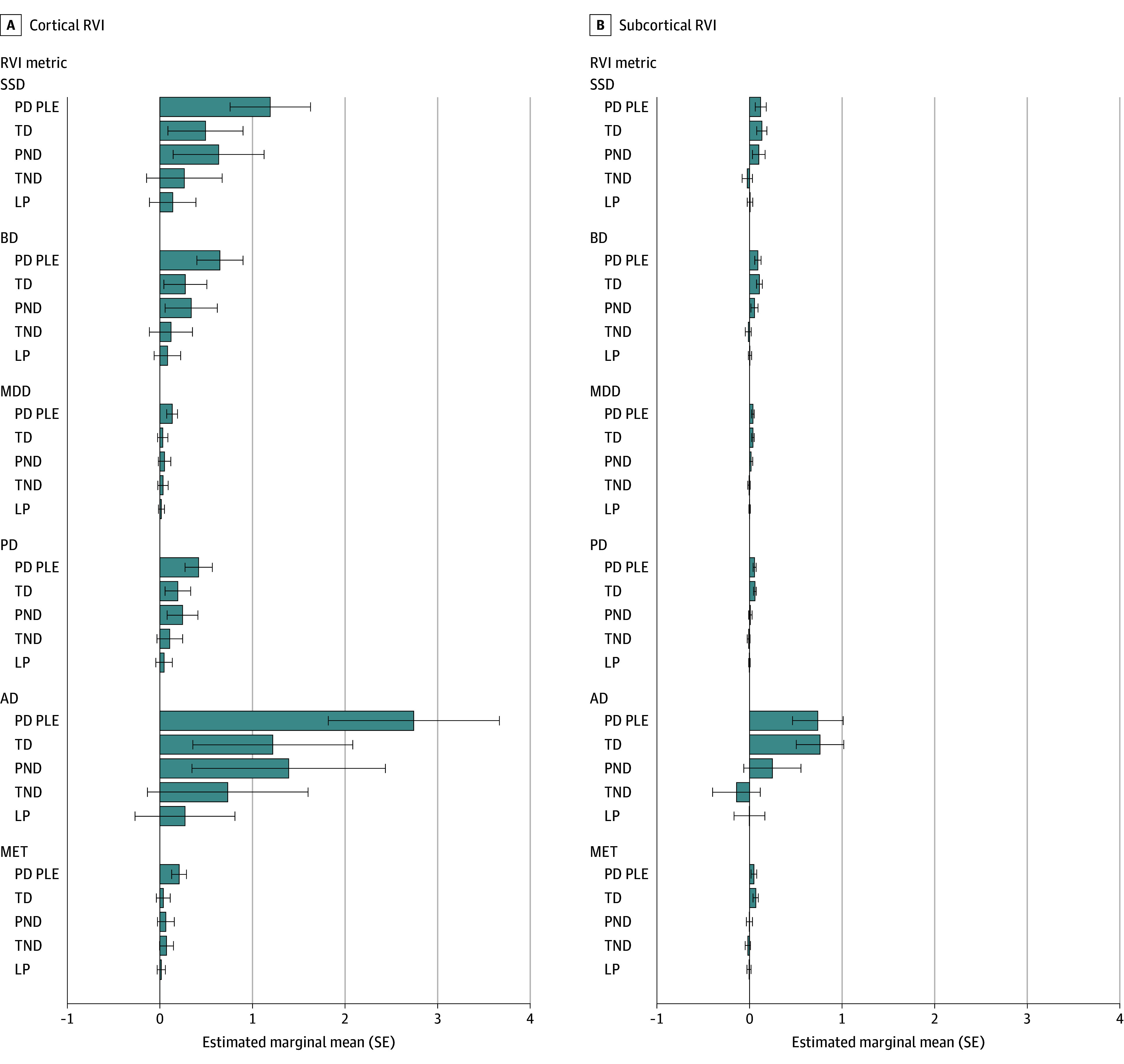
Group Comparisons for All Regional Vulnerability Index (RVI) Metrics Graphs shown for cortical (A) and subcortical (B) metrics depict estimated marginal means and SEs obtained from the linear mixed effects models for each RVI metric for each psychoticlike experience (PLE) group: persistent distressing PLEs (PD PLE), transient distressing PLEs (TD), persistent nondistressing PLEs (PND), transient nondistressing PLEs (TND), and low PLEs (LP), and for each RVI metric: schizophrenia spectrum disorder (SSD), bipolar disorder (BD), major depressive disorder (MDD), Parkinson disease (PD), Alzheimer disease (AD), and metabolic disorder (MET). During RVI metric creation, age, sex at birth, race and ethnicity, and overall intracranial volume were regressed out using linear regression. Error bars indicate SEs.

**Table 2.** Linear Mixed-Effects Model Estimates for Each PLE Group for Each Cortical and Subcortical RVI Metric^a^

RVI metric	PLE group											
	Persistent distressing			Transient distressing			Persistent nondistressing			Transient nondistressing		
	β Estimate (95% CI)	*t* Value	*P* value for FDR	β Estimate (95% CI)	*t* value	*P* value for FDR	β Estimate (95% CI)	*t* Value	*P* value for FDR	β Estimate (95% CI)	*t* Value	*P* value for FDR
**Cortical RVI**												
SSD	1.055 (0.326 to 1.786)	2.833	.04	0.354 (−0.310 to 1.020)	1.044	.55	0.497 (−0.361 to 1.356)	1.134	.55	0.127 (−0.543 to 0.798)	0.370	.80
BD	0.568 (0.150 to 0.986)	2.659	.04	0.193 (−0.188 to 0.575)	0.993	.55	0.257 (−0.234 to 0.750)	1.025	.55	0.037 (−0.347 to 0.422)	0.189	.85
MDD	0.115 (0.013 to 0.218)	2.215	.11	0.014 (−0.079 to 0.107)	0.285	.81	0.033 (−0.087 to 0.153)	0.542	.74	0.017 (−0.077 to 0.111)	0.348	.80
PD	0.374 (0.133 to 0.615)	3.040	.03	0.148 (−0.071 to 0.368)	1.324	.50	0.199 (−0.084 to 0.482)	1.375	.50	0.061 (−0.160 to 0.283)	0.543	.74
AD	2.473 (0.930 to 4.018)	3.138	.03	0.948 (−0.457 to 2.357)	1.321	.50	1.121 (−0.693 to 2.938)	1.210	.54	0.462 (−0.955 to 1.880)	0.638	.74
MET	0.192 (0.055 to 0.330)	2.741	.04	0.022 (−0.104 to 0.147)	0.336	.80	0.049 (−0.112 to 0.211)	0.597	.74	0.057 (−0.070 to 0.183)	0.880	.61
**Subcortical RVI**												
SSD	0.116 (0.010 to 0.221)	2.152	.06	0.127 (0.031 to 0.223)	2.588	.02	0.095 (−0.029 to 0.218)	1.499	.25	−0.030 (−0.126 to 0.067)	−0.599	.61
BD	0.086 (0.026 to 0.145)	2.828	.01	0.103 (0.049 to 0.157)	3.734	.001	0.049 (−0.021 to 0.119)	1.374	.29	−0.018 (−0.072 to 0.037)	−0.643	.61
MDD	0.035 (0.010 to 0.059)	2.797	.01	0.036 (0.014 to 0.058)	3.155	.006	0.017 (−0.012 to 0.045)	1.145	.40	−0.006 (−0.029 to 0.016)	−0.540	.61
PD	0.056 (0.027 to 0.085)	3.763	.001	0.059 (0.032 to 0.085)	4.345	<.001	0.010 (−0.024 to 0.044)	0.574	.61	−0.009 (−0.035 to 0.018)	−0.646	.61
AD	0.739 (0.288 to 1.189)	3.215	.005	0.762 (0.352 to 1.173)	3.641	.001	0.247 (−0.282 to 0.777)	0.916	.54	−0.141 (−0.554 to 0.273)	−0.666	.61
MET	0.052 (0.011 to 0.094)	2.456	.03	0.072 (0.034 to 0.110)	3.697	.001	0.004 (−0.045 to 0.053)	0.168	.87	−0.013 (−0.051 to 0.025)	−0.666	.61

Abbreviations: AD, Alzheimer disease; BD, bipolar disorder; FDR, false discovery rate; MDD, major depressive disorder; MET, metabolic disease; PD, Parkinson disease; PLE, psychoticlike experience; RVI, regional vulnerability index; SSD, schizophrenia spectrum disorder.

^a^ In all models, the control group served as the reference group. Unstandardized β estimates and 2-sided *t* statistics are reported. The false discovery rate was adjusted across all 24 models for cortical RVI metrics and across all 24 models for subcortical metrics.

For the transient distressing PLE group, no cortical RVI metrics were statistically significant (*t* ≤ 1.324; FDR *P* ≥ .50) (Table 2). For subcortical RVI metrics, in comparison with the low PLE group, transient distressing PLEs showed an increase in RVI for all metrics (*t* ≥ 2.588; FDR *P* < .05) (Table 2). The persistent and transient nondistressing PLE groups showed no associations for cortical or subcortical RVI metrics (*t* ≤ 1.499; FDR *P* > .24) (Table 2).

## Discussion

The similarity-based index differentiated distressing PLE groups from low PLEs in otherwise healthy children. This suggests that either the causes or consequences of distressing PLEs, and especially persistent distressing PLEs, are associated with formation of structural brain patterns with higher similarity to adults with severe psychiatric and neurologic conditions, including SSD, BD, and AD.

The ABCD Study participants with persistent and distressing PLEs showed the broadest neural deviations in the direction of disease, consistent with previous findings.^13^ Persistent distressing PLEs was the only group to show neural metrics more similar to states of disease compared with the low PLE group for cortical RVI metrics, showing the greatest effect sizes for SSD and AD risk scores. For subcortical RVI, both the persistent and transient distressing PLE groups showed evidence of having neural metrics more similar to a broad range of psychiatric, metabolic, and neurologic conditions. While the persistent distressing PLE group showed broad cortical and subcortical neural deviations in the direction of disease, the transient distressing PLE group only showed subcortical deviations in the direction of disease. It is possible that while subcortical deviations in the direction of disease develop early for those experiencing distressing PLEs, it is perhaps only with persistence that cortical deviations follow, including cortical deviations in the direction of SSD.^15^

The finding of distressing PLE groups broadly showing subcortical neural deviations in the direction of AD potentially supports the role of subcortical neurologic conditions in PLEs and is consistent with prior research investigating the link between SSD and increased risk for AD.^15^ These results were specific to PLE groups, as internalizing symptom groups did not exhibit the same pattern of findings (eTable 3 in Supplement 1). The largest RVI scores for those with distressing PLEs were for AD rather than SSDs, pointing to the potential role of neurodegenerative processes that affect limbic structures in the development of psychosis spectrum symptoms, a theory that dates back to the Kraepelin dementia praecox definition of schizophrenia.^15,16^

### Limitations

There are several limitations of the present work, including that the PLE measure is self-reported by youth and a comparable clinician-reported measure is not available. Additional work is also needed to validate this study in an external sample, although, to our knowledge, a comparable large-scale sample longitudinally assessing PLEs and associated distress as well as MRI in youth is not currently available. Additionally, the study used to derive the RVI-AD metrics had a smaller sample size compared with the other RVI metrics (n = 290 cases vs >926 cases for the other RVI metrics). ENIGMA studies aggregate findings across the world, reducing regional variation but potentially leading to concerns about whether variation in sample characteristics poses a threat to replicability. However, using this form of aggregated neuroimaging, we can identify and leverage replicable neurosignatures showing patterns of group differences.^17^ These neural signatures show up to 90% replicability in independent samples revealing robust patterns in regional patterns of disease.^8,17,18,19^

## Conclusions

In this ABCD Study cohort, the present work found initial evidence that especially the neural metrics for youth experiencing persistent distressing PLEs showed greater similarity to neural metrics for adults with chronic mental and physical health conditions, with the largest effect sizes for brain-based risk metrics for SSD and AD. Overall, the present work generates initial evidence for the role of brain-based risk scores in early identification efforts for PLEs.

## Notes

Supplement 1. **eMethods.** Additional Information on Exclusion Criteria and Measures **eTable 1.** Definitions Used to Create Psychotic-Like Experience (PLE) Groups from Prodromal Questionnaire–Brief Child Version Data from 3 Assessment Waves **eTable 2.** Characteristics of Studies Used to Calculate RVI **eTable 3.** Model Estimates for Each Internalizing Symptom Group for Each Cortical and Subcortical RVI Metric
eReferences

Supplement 2.
Data Sharing Statement
