# Error in Methods

**DOI:** 10.1001/jamanetworkopen.2025.38948

**Published:** 2025-09-24

In the Original Investigation titled “Regional Vulnerability Indices in Youth With Persistent and Distressing Psychoticlike Experiences,”^1^ published November 13, 2023, there was a wording error in the Measures portion of the Methods section. The low symptom threshold given as “(≤0.50 SDs below the mean)” should have been “(≤0.50 SDs above the mean).” This article has been corrected.^1^
