# Supplementary material for: Regional Vulnerability Indices in Youth With Persistent and Distressing Psychoticlike Experiences
Source: JAMA Netw Open. 2023 Nov 13;6(11):e2343081. doi: 10.1001/jamanetworkopen.2023.43081 (PMC10644211; doi:10.1001/jamanetworkopen.2023.43081)
Supplement: Supplement 1. — eMethods. Additional Information on Exclusion Criteria and Measures eTable 1. Definitions Used to Create Psychotic-Like Experience (PLE) Groups from Prodromal Questionnaire–Brief Child Version Data from 3 Assessment Waves eTable 2. Characteristics of Studies Used to Calculate RVI eTable 3. Model Estimates for Each Internalizing Symptom Group for Each Cortical and Subcortical RVI Metric eReferences [file jamanetwopen-e2343081-s001.pdf]

Supplementary Online Content

Karcher NR, Modi H, Kochunov P, Gao S, Barch DM. Regional vulnerability indices in youth with persistent and distressing psychoticlike experiences. *JAMA Netw Open*. 2023;6(11):e2343081. doi:10.1001/jamanetworkopen.2023.43081

eMethods. Additional Information on Exclusion Criteria and Measures

eTable 1. Definitions Used to Create Psychotic-Like Experience (PLE) Groups from Prodromal Questionnaire–Brief Child Version Data from 3 Assessment Waves

eTable 2. Characteristics of Studies Used to Calculate RVI

eTable 3. Model Estimates for Each Internalizing Symptom Group for Each Cortical and Subcortical RVI Metric

eReferences

This supplementary material has been provided by the authors to give readers additional information about their work.

© 2023 Karcher NR et al. *JAMA Network Open.*

eMethods. Additional Information on Exclusion Criteria and Measures

Participants

Potential participants were excluded for the following reasons: child not fluent in English, MRI contraindication (e.g., irremovable ferromagnetic implants or dental appliances, claustrophobia, pregnant), major neurological disorder, gestational age less than 28 weeks or birthweight less than 1,200 grams, history of traumatic brain injury, or had a current diagnosis of schizophrenia, autism spectrum disorder (moderate, severe), mental retardation/intellectual disability, or alcohol/substance use disorder.

Measures

Brief Problem Monitor

The present study also utilized the internalizing scale raw scores from an abbreviated form of the Youth Self Report, the youth-rated Brief Problem Monitor (BPM)[1] to create internalizing symptom groups. The BPM asks youth to rate 19 items assessing current psychopathology (i.e., within the past six months) on a 0 (not true) to 2 (very true or often true) scale. To create internalizing symptom groups we utilized the three waves of BPM scores (6-month follow-up, 1-year follow-up, 2-year follow-up). Persistent and transient internalizing symptom groups were created using the same definitions as used for PLEs: the persistent internalizing group (n=232) met criteria for the elevated internalizing score threshold (i.e., >=1.96 SDs above the mean BPM score) for 2 or more of the three waves of data collection; the transient internalizing group (n=183) met criteria for the elevated internalizing score threshold (i.e., >=1.96 SDs above the mean BPM score) for 1 wave of data collection and met criteria for the low internalizing score threshold (i.e., <=0.50 SDs below the mean BPM Score) for the other 2 waves. See eTable 2 for results using these internalizing symptom groups. Reference group was a low-level internalizing symptom group who met the low internalizing score threshold (i.e., <=0.50 SDs below the mean BPM Score) for all 3 waves.

© 2023 Karcher NR et al. *JAMA Network Open.*

eTable 1. Definitions used to Create Psychotic-Like Experience (PLE) Groups from Prodromal Questionnaire-Brief Child Version Data from 3 Assessment Waves[a]

	Persistent Distressing (n=329)	Transient Distressing (n=396)	Persistent Non-Distressing (n=234)	Transient Non-Distressing (n=390)	Low PLEs (n=6893)
PLE Score Used	Distress Score	Distress Score	Non-Distress Score	Non-Distress Score	Distress and Non-Distress Scores
Number of Waves Meeting Elevated PLE Threshold (i.e., >=1.96 SDs above the mean PLE Score)	≥2	1	≥2	1	0
Number of Waves Meeting Low PLE Threshold (i.e., <=0.50 SDs below the mean PLE Score)	≤1	2	≤1	2	3

[a]See Table 1 for sample demographic characteristics.

© 2023 Karcher NR et al. *JAMA Network Open.*

eTable 2. Characteristics of Studies used to Calculate RVI

RVI Phenotype	Cortical vs. Subcortical	Citation	Sample Size
SSD	Cortical	van Erp et al., 2018[2]	9,572 (4474 SSD/5098 controls)
	Subcortical	van Erp et al., 2016[3]	8,927 (1728 MDD/7199 controls)
BD	Cortical	Hibar et al., 2018[4]	6503 (1837 BD/ 2582 controls)
	Subcortical	Hibar et al., 2016[5]	4,304 (1710 BD/ 2594 controls)
MDD	Cortical	Schmaal et al., 2017[6]	10,105 (2148 MDD/7957 controls)
	Subcortical	Schmaal et al., 2016[7]	8,927 (1728 MDD/7199 controls)
PD	Cortical & Subcortical	Laansma et al., 2021[8]	3,539 (2,357 PD/1182 controls)
AD	Cortical & Subcortical	Kochunov et al., 2021[9]	898 (290 AD/608 controls)
MET	Cortical & Subcortical	Hatch et al, 2023[10]	6,420 (926 MET/2666 controls)

Abbreviations. RVI=regional vulnerability index; SSD=schizophrenia spectrum disorder; BD=bipolar disorder; MDD=major depressive disorder; PD=Parkinson's disease; AD=Alzheimer's disease; MET=metabolic disease.

© 2023 Karcher NR et al. *JAMA Network Open.*

eTable 3. Model Estimates for Each Internalizing Symptom Group[a] for each Cortical and Subcortical RVI Metric

	Persistent Internalizing (n=232)				Transient Internalizing (n=183)			
	Est.	SE	*t*	*p*	Est.	SE	*t*	*p*
**Cortical RVI**								
SSD	-0.328	0.438	-0.748	.45	-0.217	0.490	-0.442	.66
BD	-0.200	0.251	-0.796	.43	-0.135	0.280	-0.480	.63
MDD	-0.082	0.061	-1.337	.18	-0.041	0.068	-0.606	.54
PD	-0.106	0.145	-0.732	.46	-0.022	0.162	-0.135	.89
AD	-0.757	0.927	-0.817	.41	-0.291	1.037	-0.280	.78
MET	-0.107	0.082	-1.301	.19	-0.093	0.092	-1.010	.31
**Subcortical RVI**								
SSD	0.104	0.064	1.625	.10	-0.006	0.071	-0.090	.93
BD	0.083	0.036	2.322	.02	-0.001	0.040	-0.026	.98
MDD	0.033	0.015	2.210	.03	-0.008	0.016	-0.502	.62
PD	0.051	0.018	2.921	.004	0.007	0.020	0.349	.73
AD	-0.757	0.927	-0.817	.41	-0.291	1.037	-0.280	.78
MET	0.054	0.025	2.137	.03	-0.020	0.028	-0.706	.48

Abbreviations. Est.=Unstandardized beta estimate; SE=standard error; t=two-sided t statistic; p=p-value; RVI=regional vulnerability index; SSD=schizophrenia spectrum disorder; BD=bipolar disorder; MDD=major depressive disorder; PD=Parkinson's disease; AD=Alzheimer's disease; MET=metabolic disease.

[a]Persistent internalizing group met criteria for the elevated internalizing score threshold (i.e., 1.96 SDs above the mean of the BPM score) for 2 or more of the three waves of data collection; the transient internalizing group met criteria for the elevated internalizing score threshold (i.e., 1.96 SDs above the mean of the BPM score) for 1 wave of data collection and met criteria for the low internalizing score threshold (i.e., 0.50 SDs above the mean of the BPM Score) for the other 2 waves. Reference group was a low-level internalizing symptom group who met the low internalizing score threshold (i.e., 0.50 SDs above the mean of the BPM Score) for all 3 waves.

© 2023 Karcher NR et al. *JAMA Network Open.*

eReferences

1. Achenbach TM, McConaughy SH, Ivanova MY, Rescorla LA. Manual for the ASEBA brief problem monitor (BPM). *Burlington, VT: ASEBA*. Published online 2011:1-33.

2. van Erp TGM, Walton E, Hibar DP, et al. Cortical Brain Abnormalities in 4474 Individuals With Schizophrenia and 5098 Control Subjects via the Enhancing Neuro Imaging Genetics Through Meta Analysis (ENIGMA) Consortium. *Biol Psychiatry*. Published online 2018. doi:10.1016/j.biopsych.2018.04.023

3. Van Erp TGM, Hibar DP, Rasmussen JM, et al. Subcortical brain volume abnormalities in 2028 individuals with schizophrenia and 2540 healthy controls via the ENIGMA consortium. *Molecular Psychiatry 2016 21:4*. 2015;21(4):547-553. doi:10.1038/mp.2015.63

4. Hibar D, Westlye L, Doan N, … NJM, 2018 undefined. Cortical abnormalities in bipolar disorder: an MRI analysis of 6503 individuals from the ENIGMA Bipolar Disorder Working Group. *nature.comDP Hibar, LT Westlye, NT Doan, N Jahanshad, JW Cheung, CRK Ching, A VersaceMolecular psychiatry, 2018•nature.com*. Accessed September 19, 2023. https://www.nature.com/articles/mp201773

5. Hibar DP, Westlye LT, Van Erp TGM, et al. Subcortical volumetric abnormalities in bipolar disorder. *Molecular Psychiatry 2016 21:12*. 2016;21(12):1710-1716. doi:10.1038/mp.2015.227

6. Schmaal L, Hibar DP, Sämann PG, et al. Cortical abnormalities in adults and adolescents with major depression based on brain scans from 20 cohorts worldwide in the ENIGMA Major Depressive Disorder Working Group. *Mol Psychiatry*. 2017;22(6):900. doi:10.1038/MP.2016.60

7. Schmaal L, Veltman DJ, Van Erp TGM, et al. Subcortical brain alterations in major depressive disorder: findings from the ENIGMA Major Depressive Disorder working group. *Molecular Psychiatry 2016 21:6*. 2015;21(6):806-812. doi:10.1038/mp.2015.69

8. Laansma MA, Bright JK, Al-Bachari S, et al. International Multicenter Analysis of Brain Structure Across Clinical Stages of Parkinson's Disease. *Mov Disord*. 2021;36(11):2583-2594. doi:10.1002/MDS.28706

9. Kochunov P, Ryan MC, Yang Q, et al. Comparison of regional brain deficit patterns in common psychiatric and neurological disorders as revealed by big data. *Neuroimage Clin*. 2021;29. doi:10.1016/J.NICL.2021.102574

10. Hatch KS, Gao S, Ma Y, et al. Brain deficit patterns of metabolic illnesses overlap with those for major depressive disorder: A new metric of brain metabolic disease. *Hum Brain Mapp*. 2023;44(6):2636-2653. doi:10.1002/HBM.26235

© 2023 Karcher NR et al. *JAMA Network Open.*
