# Supplementary material for: Regional Vulnerability Indices in Youth With Persistent and Distressing Psychoticlike Experiences
Source: JAMA Netw Open. 2023 Nov 13;6(11):e2343081. doi: 10.1001/jamanetworkopen.2023.43081 (PMC10644211; doi:10.1001/jamanetworkopen.2023.43081)
Supplement: Supplement 2. — Data Sharing Statement [file jamanetwopen-e2343081-s002.pdf]

Data Sharing Statement

Karcher. Regional Vulnerability Indices in Youth With Persistent and Distressing Psychoticlike Experiences. *JAMA Netw Open*. Published November 13, 2023.
doi:10.1001/jamanetworkopen.2023.43081

Data
Data available: Yes
Data types: Deidentified participant data
How to access data: Data are available through the NDA: https://nda.nih.gov
When available: With publication

Supporting Documents
Document types: None

Additional Information
Who can access the data: Anyone with an approved data use agreement can assess the data.
Types of analyses: Anyone with an approved data use agreement can assess the data, regardless of type of analysis.
Mechanisms of data availability: Data will be made available for anyone with an approved data use agreement.
